# Body Weight Variability and Risk of Cardiovascular Outcomes and Death in the Context of Weight Loss Intervention Among Patients With Type 2 Diabetes

**DOI:** 10.1001/jamanetworkopen.2022.0055

**Published:** 2022-02-18

**Authors:** Arnaud D. Kaze, Prasanna Santhanam, Sebhat Erqou, Rexford S. Ahima, Alain G. Bertoni, Justin B. Echouffo-Tcheugui

**Affiliations:** 1 Department of Medicine, SOVAH Health, Danville, Maryland; 2 Division of Endocrinology, Diabetes & Metabolism, Department of Medicine, Johns Hopkins School of Medicine, Baltimore, Maryland; 3 Department of Medicine, Providence VA Medical Center, Providence, Rhode Island; 4 Alpert Medical School, Brown University, Providence, Rhode Island; 5 Department of Epidemiology and Prevention, Wake Forest School of Medicine, Winston-Salem, North Carolina

## Abstract

**Importance:**

Body weight fluctuation is associated with greater risks of adverse health outcomes. Whether intensive weight loss interventions affect the association of variability in adiposity measures with adverse health outcomes in individuals with type 2 diabetes has not been studied previously.

**Objective:**

To evaluate the associations of long-term variability in adiposity indices with cardiovascular disease (CVD) outcomes and whether these associations are affected by an intensive lifestyle intervention among adults with type 2 diabetes.

**Design, Setting, and Participants:**

This prospective cohort study included participants in the Action for Health in Diabetes (Look AHEAD) trial without CVD at baseline (August 2001 to April 2004). The Look AHEAD study included 16 centers in the United States. Data analysis was performed from December 2020 to June 2021.

**Exposures:**

Variability of body mass index (BMI) and waist circumference (WC) across 4 annual visits, assessed using the coefficient of variation (CV), variability independent of the mean (VIM), and standard deviation (SD).

**Main Outcomes and Measures:**

Main outcomes were (1) all-cause mortality, (2) cardiovascular deaths (deaths from myocardial infarction [MI] or stroke), and (3) CVD events (MI, stroke, and/or death from cardiovascular causes).

**Results:**

Among 3604 study participants (mean [SD] age, 58.4 [6.6] years; 2240 [62.3%] women; 1364 [37.7%] Black participants; 2404 [66%] White participants), there were 216 CVD events, 33 CVD deaths, and 166 deaths over a median of 6.7 years. In the control group, the hazard ratios (HRs) for the highest quartile (quartile 4) compared with the lowest quartile (quartile 1) of CV of BMI were 4.06 (95% CI, 2.17-7.57), 15.28 (95% CI, 2.89-80.90), and 2.16 (95% CI, 1.21-3.87) for all-cause mortality, CVD mortality, and cardiovascular events, respectively. In the intervention group, the corresponding HRs were 0.99 (95% CI, 0.45-2.16), 1.14 (95% CI, 0.12-10.53), and 0.77 (95% CI, 0.40-1.49) for quartile 4 vs quartile 1. Regarding WC, in the control group, HRs for quartile 4 vs quartile 1 were 1.84 (95% CI, 1.01-3.35), 6.46 (95% CI, 1.16-36.01), and 1.28 (95% CI, 0.72-2.29). In the intervention group, HRs were 1.23 (95% CI, 0.61-2.46), 0.55 (95% CI, 0.15-2.11), and 0.70 (95% CI, 0.39-1.25) for quartile 4 vs quartile 1.

**Conclusions and Relevance:**

In this cohort study of individuals with type 2 diabetes, higher variability of adiposity indices was associated with significantly increased risk of CVD outcomes and death in the control group but not in the intensive lifestyle intervention group.

## Key Points

**Question:**

How does an intensive lifestyle intervention affect the association between variability in adiposity indices and cardiovascular outcomes and deaths among adults with type 2 diabetes?

**Findings:**

In this cohort study of 3604 adults with type 2 diabetes, a greater variability in body mass index and waist circumference was independently associated with higher risks of cardiovascular events, cardiovascular mortality, and all-cause mortality in the control group. In the intensive lifestyle intervention group, no association was observed between variability in these indices and the outcomes.

**Meaning:**

These findings suggest that weight fluctuations during weight loss attempts may lead to increased cardiovascular and mortality risk among individuals with type 2 diabetes.

## Teaser

This cohort study evaluates the associations of long-term variability in adiposity indices with cardiovascular disease (CVD) outcomes and whether these associations are affected by an intensive lifestyle intervention among adults with type 2 diabetes.

## Introduction

Type 2 diabetes and obesity are common in the United States.^1,2^ Obesity is associated with the development of type 2 diaestes.^3^ Obesity management is associated with improved outcomes in individuals with type 2 diabetes.^4^ Accordingly, the American Diabetes Association recommends weight loss interventions for patients with overweight or obesity and type 2 diabetes.^4^ Lifestyle interventions frequently lead to transient weight loss; with repeated and interrupted attempts often resulting in significant weight fluctuations—a process referred to as weight cycling.^5^ Body weight fluctuations are associated with greater risks of adverse health outcomes, independent of the extent of obesity.^5,6^

While a few studies have evaluated the association of body weight variability with cardiovascular disease (CVD) outcomes and deaths, data among people with type 2 diabetes are sparse.^7,8,9,10,11^ Furthermore, these studies have typically focused on the variability of body mass index (BMI; calculated as weight in kilograms divided by height in meters squared), a measure of overall adiposity, and did not assess the variability of markers of abdominal obesity, such as waist circumference (WC). BMI captures both fat and lean masses, whereas WC may be more specific to adiposity. Furthermore, abdominal obesity assessed by WC was associated with adverse cardiovascular outcomes while general obesity (measured by BMI) without abdominal obesity was not.^12^ Additionally, to our knowledge, how intensive weight loss interventions affect the association of variability in adiposity measures with adverse health outcomes in individuals with type 2 diabetes has not been studied previously. The Action for Health in Diabetes (Look AHEAD) study included an intensive lifestyle intervention (ILI) to achieve weight loss, thus providing a unique opportunity to address such a research question.^13^

We conducted a cohort analysis of Look AHEAD data to evaluate the associations of long-term variability in 2 adiposity indices (BMI and WC) with incident cardiovascular outcomes and mortality and assessed whether these associations were affected by lifestyle interventions aiming to achieve weight loss among individuals with type 2 diabetes.

## Methods

### Study Design

This report followed the Strengthening the Reporting of Observational Studies in Epidemiology (STROBE) reporting guideline for observational studies.^14^ The design and methods of Look AHEAD have been published.^13^ Each participant provided informed consent, and the study proposal was approved by the institutional review board at each participating location.^13^ This study did not seek additional institutional review board approval. Briefly, Look AHEAD was a multicenter, randomized clinical trial that evaluated the effects of an ILI compared with the standard of care (diabetes support and education [DSE]) on cardiovascular outcomes in individuals with overweight or obesity and type 2 diabetes. A total of 5145 participants aged 45 to 76 years were enrolled from August 2001 to April 2004 across 16 locations in the United States. The trial was terminated on September 14, 2012.^15^

The randomization groups included an ILI group (intervention group) and a DSE group (control group). The ILI aimed to achieve a 7% weight loss or greater via increased physical activity and decreased caloric intake. The intervention included both individual and group counseling sessions, occurring weekly for the first 6 months, then with reducing frequency throughout the rest of the trial. The DSE included 3 group sessions per year focused on exercise, diet, and social support throughout the first 4 years, then 1 session yearly throughout the rest of the study.

The current analysis was restricted to participants with complete data on BMI and WC at the baseline, 12-, 24-, and 36-month visits. Participants with prevalent CVD at baseline (n = 691) or with consent restrictions (n = 244) were excluded. To ensure that the exposure preceded the development of outcomes, we also excluded individuals who developed CVD events or died during the variability assessment period, which is the initial 36 months following the start of the study. After these exclusions, a total of 3604 participants were included in our analyses (eFigure in the Supplement).

### Assessment of Variability of Adiposity Measures

At each visit, participants’ weight and height were measured twice using a digital scale and a standard stadiometer, respectively; and the average of those measurements were used for the analyses. WC was measured with participants in light clothing using a nonmetallic, constant tension tape placed around the body at midlevel between the highest point of the iliac crest and lowest point of the costal margin on the midaxillary line.^13,15,16^

For each adiposity measure (BMI, WC, or body weight), long-term variability was defined using 3 metrics: (1) the coefficient of variation (CV); (2) the intra-individual standard deviation (SD); and (3) the variability independent of the mean (VIM) computed as 100 × SD / mean^β^, where β is the regression coefficient based on the natural logarithm of SD on the natural logarithm of the obesity measure’s mean.^17^ Higher values for CV, VIM or SD indicate a greater variability.

### Ascertainment of Incident Clinical Outcomes

We evaluated 3 outcomes: (1) all-cause mortality; (2) cardiovascular deaths (deaths from myocardial infarction or stroke); and (3) CVD events (composite of myocardial infarction, stroke, and death from cardiovascular causes). The participants were prospectively followed from the end of the variability assessment period until the occurrence of an outcome or the end of study, whichever came first. The details of the ascertainment and adjudication of outcomes in Look AHEAD have been published and are provided in eMethods in the Supplement.^13^

### Covariates

The selection of covariates included in regression models was performed a priori based on evidence on their role as potential confounders. The covariates selected at baseline included age, sex, race and ethnicity (self-reported by participants), randomization arm, smoking status, alcohol consumption, duration of diabetes, and estimated glomerular filtration rate (eGFR) calculated using the Chronic Kidney Disease–Epidemiology Collaboration equation.^18^ Racial and ethnic groups included American Indian or Alaska Native, Asian or Pacific Islander, Black, Hispanic, White, biracial, other, and unknown. Individuals selecting American Indian or Alaska Native, Asian or Pacific Islander, biracial, other, or unknown were classified as other race and ethnicity. The study group covariates measured throughout the variability assessment period included mean hemoglobin A_1c_ (HbA_1c_), mean ratio of total to high-density lipoprotein (HDL) cholesterol, mean systolic and diastolic blood pressures (BPs), and use of BP-lowering medication.^13^

### Statistical Analysis

We tested and found a significant interaction between BMI variability measures and randomization group for all-cause mortality . For this reason, the clinical relevance of examining all outcomes by treatment arms, and the need for consistency in reporting our results, all our analyses are stratified by randomization groups. Characteristics of participants were reported across quartiles of CV of each adiposity marker as mean (SD) or median (IQR) for continuous variables or proportions for categorical variables. The quartiles were calculated based on the pooled data from both groups. The comparison of categorical variables was performed using the χ^2^ test, whereas continuous variables were compared using analysis of variance or Kruskal-Wallis tests.

Cox proportional hazards regression models were used to calculate hazard ratios (HRs) and associated 95% CIs for each outcome. Regarding missing data, we did not conduct any imputation. We conducted complete case analyses. The participants lost to follow-up were censored at the time of last visit. Each variability index was assessed both as continuous and categorical (quartile) variables. Three nested models were constructed for each variability marker. Model 1 adjusted for age, sex, race, and ethnicity. Model 2 included model 1 plus adjustment for current smoking, alcohol drinking, use of BP-lowering medication, mean total-to-HDL cholesterol ratio, eGFR, mean systolic BP, mean HbA_1c_ level, and duration of diabetes. Model 3 adjusted for variables in model 2 with further adjustment for mean BMI (when assessing the variability of BMI), mean WC (in models evaluating the variability of WC), or mean weight (when assessing weight variability). All analyses were stratified by trial arm (DSE or ILI).

To further assess the association of large weight changes with our findings, we conducted additional adjustments for BMI change (or WC change) between the baseline and fourth visits. We also performed analyses restricting the sample to individuals for whom weight change between the baseline and fourth visits was less than 5 kg.

All statistical analyses were performing using Stata 14.2 (StataCorp). A 2-sided *P* < .05 was considered statistically significant.

## Results

### Characteristics of Study Participants

The study sample consisted of 3604 participants (mean [SD] age 58.4 [6.6] years; 2240 [62.3%] women, 1364 [37.7%] men). A comparison of the baseline characteristics of the included participants with those excluded from the final sample is provided in eTable 1 in the Supplement. The baseline characteristics of participants by study group and quartiles of CV of BMI are displayed in Table 1. In each study group, compared with the lowest quartile (quartile 1), participants in the highest quartile (quartile 4) were more likely to be White (DSE: 159 of 213 [74.7%] vs 445 of 684 [65.1%]; ILI: 506 of 688 [73.6%] vs 112 of 217 [51.6%]). Among participants in the DSE group, those in quartile 4 had higher mean (SD) BMI values than those in quartile 1 (36.4 [6.2] vs 35.4 [5.5]) over the variability assessment period; whereas in the ILI group, participants in quartile 4 were more likely to have lower mean (SD) BMI than those in quartile 1 (33.0 [5.7] vs 35.5 [6.1]).

**Table 1.** Characteristics of Study Participants by Quartiles of BMI Variability Stratified by Treatment Group in the Look Action for Health in Diabetes Study

Characteristic	Participants in DSE group, by quartile of coefficient of variation of BMI, No. (%) (n = 1775)					Participants in ILI group, by quartile of coefficient of variation of BMI, No. (%) (n = 1829)							
	1, <1.97% (n = 684)	2, 1.97%-3.15% (n = 534)	3, 3.15%-4.94% (n = 344)	4, >4.94% (n = 213)	*P* value	1, <1.97% (n = 217	2, 1.97%-3.15% (n = 367)		3, 3.15%-4.94% (n = 557)		4, >4.94% (n = 688)		*P* value
**At baseline**													
Age, mean (SD), y	58.9 (6.8)	58.7 (6.5)	58.2 (6.6)	58.1 (7.1)	.22	57.5 (6.5)	57.4 (6.1)		58.7 (6.8)		58.6 (6.7)		.006
Sex													
Women	384 (56.1)	337 (63.1)	225 (65.4)	155 (72.8)	<.001	128 (59.0)	242 (65.9)		366 (65.7)		403 (58.6)		.02
Men	300 (43.9)	197 (36.9)	119 (34.6)	58 (27.2)		89 (41.0)	125 (34.1)		191 (34.3)		285 (41.4)		
Race and ethnicity													
Black	131 (19.2)	101 (18.9)	57 (16.6)	23 (10.8)	.03	61 (28.1)	77 (21.0)		102 (18.3)		76 (11.1)		<.001
Hispanic	86 (12.6)	78 (14.6)	30 (8.7)	26 (12.2)		32 (14.8)	46 (12.5)		63 (11.3)		90 (13.1)		
White	445 (65.1)	337 (63.1)	244 (70.9)	159 (74.7)		112 (51.6)	230 (62.7)		371 (66.6)		506 (73.6)		
Other^a^	22 (3.2)	18 (3.4)	13 (3.8)	5 (2.4)		12 (5.5)	14 (3.8)		21 (3.8)		16 (2.3)		
Current smoking	18 (2.6)	21 (3.9)	9 (2.6)	9 (4.2)	.44	8 (3.7)	21 (5.7)		21 (3.8)		24 (3.5)		.34
Alcohol drinking	247 (36.1)	183 (34.3)	107 (31.1)	59 (27.7)	.10	78 (35.9)	131 (35.7)		188 (33.8)		215 (31.3)		.40
Diabetes duration, median (IQR), y	5.0 (2.0-10.0)	5.0 (2.0-9.0)	4.5 (2.0-8.0)	5.0 (2.0-9.0)	.21	5.0 (2.0-9.0)	4.0 (2.0-9.0)		5.0 (2.0-10.0)		5.0 (2.0-9.0)		.79
eGFR, mean (SD), mL/min/1.73 m^2^	91.4 (14.7)	90.2 (15.8)	90.2 (16.3)	90.9 (16.0)	.49	92.1 (16.3)	92.0 (15.7)		91.5 (15.9)		89.7 (15.8)		.05
**During variability assessment period**													
Hemoglobin A_1c_, mean (SD), %	7.2 (1.0)	7.1 (1.0)	7.1 (1.1)	6.9 (1.0)	.01	7.3 (1.1)		7.2 (1.0)		6.9 (0.9)		6.5 (0.9)	<.001
Total-to-HDL cholesterol ratio, mean (SD)	4.4 (1.2)	4.4 (1.2)	4.1 (1.2)	4.0 (1.1)	<.001	4.4 (1.2)		4.4 (1.3)		4.2 (1.2)		4.0 (1.1)	<.001
Use of BP-lowering drug, %	557 (84.3)	441 (85.0)	277 (82.4)	177 (85.5)	.73	175 (84.1)		284 (79.1)		453 (82.8)		556 (83.1)	.34
Systolic BP, mean (SD), mm Hg	127.6 (12.8)	127.8 (14.3)	126.7 (13.2)	124.6 (13.5)	.02	125.7 (13.9)		126.1 (13.9)		124.2 (13.8)		122.5 (14.6)	<.001
Diastolic BP, mean (SD), mm Hg	69.7 (7.9)	69.1 (8.0)	68.1 (7.5)	66.1 (7.5)	<.001	69.8 (8.2)		69.2 (8.4)		67.8 (7.9)		67.2 (7.8)	<.001
BMI, mean (SD)	35.4 (5.5)	35.9 (5.7)	36.2 (6.0)	36.4 (6.2)	.04	35.5 (6.1)		35.1 (6.2)		34.0 (5.8)		33.0 (5.7)	<.001
WC, mean (SD), cm	112.6 (12.6)	113.0 (13.3)	113.6 (13.4)	112.9 (14.2)	.72	112.8 (13.4)		110.7 (13.5)		108.9 (13.8)		106.6 (13.7)	<.001

Abbreviations: BMI, body mass index (calculated as weight in kilograms divided by height in meters squared); BP, blood pressure; DSE, diabetes support and education; eGFR, estimated glomerular filtration rate; HDL, high-density lipoprotein; ILI, intensive lifestyle intervention; WC, waist circumference.

SI conversion factor: To convert hemoglobin to proportion of total hemoglobin, multiply by 0.01.

^a^ Race and ethnicity information were self-reported by participants. Other race and ethnicity includes race and ethnicity indicated specifically as Asian or Pacific Islander, American Indian or Alaska Native, biracial, other, or unknown.

The characteristics of participants by study group and quartiles of CV of WC are shown in eTable 2 in the Supplement. Over a median (IQR) follow-up 6.7 (6.0-7.4) years, there were 216 CVD events, 33 CVD deaths, and 166 deaths.

### Variability of BMI and Cardiovascular Outcomes

#### DSE Group

The HRs by CV of BMI are displayed in Table 2. Among participants in the DSE group, the HRs for each 3% (ie, 1-SD) increment in CV of BMI were 1.47 (95% CI, 1.24-1.74) for all-cause mortality, 1.78 (95% CI, 1.26-2.53) for CVD mortality, and 1.19 (95% CI, 0.97-1.45) for CVD events. When analyzed as quartiles, participants in quartile 4 had increased hazards for all-cause mortality (HR, 4.06 [95% CI, 2.17-7.57]), CVD-related mortality (HR, 15.28 [95% CI, 2.89-80.90]), and CVD events (HR, 2.16 [95% CI, 1.21-3.87]) compared with those in quartile 1.

**Table 2.** Hazard Ratios for Study Outcomes by Coefficient of Variation of BMI Stratified by Treatment Group in the Action for Health in Diabetes Study

Outcome	Quartiles of coefficient of variation of BMI				*P* for trend	Per 3% increase
	1, <1.97%	2, 1.97%-3.15%	3, 3.15%-4.94%	4, >4.94%		
**Diabetes support and education group (n = 1775)**						
All-cause mortality						
No. of events/person-years	20/4561.8	30/3510.7	16/2239.8	21/1385.3	NA	87/11 697.6
Rate per 1000 person-years (95% CI)	4.4 (2.8-6.8)	8.5 (6.0-12.2)	6.7 (4.0-11.1)	15.2 (9.9-23.2)		7.4 (6.0-9.1)
Hazard ratio (95% CI)^a^						
Model 1	1 [Reference]	2.18 (1.23-3.84)	1.77 (0.91-3.47	4.19 (2.26-7.77)	<.001	1.45 (1.23-1.71)
Model 2	1 [Reference]	2.05 (1.16-3.63)	1.81 (0.92-3.57)	4.18 (2.24-7.77)	<.001	1.48 (1.25-1.75)
Model 3	1 [Reference]	2.03 (1.15-3.60)	1.79 (0.91-3.52)	4.06 (2.17-7.57)	<.001	1.47 (1.24-1.74)
Cardiovascular death						
No. of events/person-years	2/4561.6	2/3510.7	4/2239.1	6/1385.3	NA	14/11 696.7
Rate per 1000 person-years (95% CI)	0.4 (0.1-1.8)	0.3 (0.0-2.0)	1.8 (0.7-4.8)	4.3 (1.9-9.6)	NA	1.1 (0.6-1.9)
Hazard ratio (95% CI)^a^						
Model 1	1 [Reference]	0.72 (0.07-7.94)	4.57 (0.83-25.07)	12.55 (2.50-63.11)	<.001	1.58 (1.14-2.18)
Model 2	1 [Reference]	0.57 (0.05-6.33)	3.59 (0.63-20.46)	11.71 (2.29-59.96)	.001	1.70 (1.19-2.43)
Model 3	1 [Reference]	0.56 (0.05-6.28)	3.70 (0.65-21.05)	15.28 (2.89-80.90)	<.001	1.78 (1.26-2.53)
Cardiovascular events						
No. of events/person-years	35/4447.9	39/3397.2	19/2189.4	19/1337.6	NA	112/11 372.1
Rate per 1000 person-years (95% CI)	7.9 (5.6-11.0)	11.5 (8.4-15.7)	8.7 (5.5-13.6)	13.5 (8.5-21.4)	NA	9.8 (8.1-11.8)
Hazard ratio (95% CI)^a^						
Model 1	1 [Reference]	1.55 (0.98-2.44)	1.18 (0.67-2.06)	1.90 (1.07-3.37)	.07	1.12 (0.92-1.37)
Model 2	1 [Reference]	1.48 (0.93-2.36)	1.22 (0.69-2.14)	2.11 (1.18-3.76)	.03	1.18 (0.96-1.44)
Model 3	1 [Reference]	1.49 (0.94-2.38)	1.23 (0.70-2.16)	2.16 (1.21-3.87)	.03	1.19 (0.97-1.45)
**Intensive lifestyle intervention group (n = 1829)**						
All-cause mortality						
No. of events/person-years	10/1428.0	14/2413.7	24/3711.6	31/4512.9	NA	79/12 066.1
Rate per 1000 person-years (95% CI)	7.0 (3.8-13.0)	5.8 (3.4-9.8)	6.5 (4.3-9.6)	6.9 (4.8-9.8)	NA	6.5 (5.3-8.2)
Hazard ratio (95% CI)^a^						
Model 1	1 [Reference]	0.84 (0.37-1.90)	0.75 (0.35-1.58)	0.84 (0.41-1.73)	.72	1.11 (0.91-1.35)
Model 2	1 [Reference]	0.82 (0.35-1.93)	0.75 (0.34-1.66)	1.00 (0.46-2.18)	.82	1.23 (1.02-1.50)
Model 3	1 [Reference]	0.82 (0.35-1.92)	0.74 (0.33-1.65)	0.99 (0.45-2.16)	.84	1.23 (1.01-1.50)
Cardiovascular death						
No. of events/person-years	1/1428.0	4/2413.7	6/3711.2	8/4512.9	NA	19/12 065.8
Rate per 1000 person-years (95% CI)	0.7 (0.1-5.0)	1.2 (0.4-3.9)	1.6 (0.7-3.6)	1.8 (0.9-3.5)	NA	1.5 (0.9-2.4)
Hazard ratio (95% CI)^a^						
Model 1	1 [Reference]	1.77 (0.18-17.10)	1.86 (0.22-15.62)	2.03 (0.25-16.40)	.55	1.32 (0.97-1.79)
Model 2	1 [Reference]	1.46 (0.15-14.28)	0.93 (0.10-8.73)	1.32 (0.15-12.04)	.90	1.55 (1.09-2.20)
Model 3	1 [Reference]	1.36 (0.14-13.36)	0.84 (0.09-7.92)	1.14 (0.12-10.53)	.98	1.54 (1.05-2.25)
Cardiovascular events						
No. of events/person-years	18/1386.1	20/2367.1	32/3647.0	34/4430.8	NA	104/11 831.0
Rate 1000 person-years (95% CI)	12.3 (7.6-19.7)	8.4 (5.5-13.1)	8.8 (6.2-12.4)	7.7 (5.5-10.7)	NA	8.7 (7.2-10.6)
Hazard ratio (95% CI)^a^						
Model 1	1 [Reference]	0.74 (0.38-1.41)	0.68 (0.37-1.23)	0.60 (0.33-1.08)	.11	0.93 (0.76-1.14)
Model 2	1 [Reference]	0.76 (0.38-1.53)	0.76 (0.40-1.47)	0.79 (0.41-1.52)	.61	1.07 (0.87-1.31)
Model 3	1 [Reference]	0.76 (0.38-1.52)	0.75 (0.39-1.44)	0.77 (0.40-1.49)	.56	1.06 (0.86-1.31)

Abbreviations: BMI, body mass index; NA, not applicable.

^a^ Model 1 adjusted for age, sex, race and ethnicity. Model 2 includes variables in model 1 with further adjustment for current smoking, alcohol drinking, use of antihypertensive medications, mean systolic blood pressure, mean ratio of total to high-density lipoprotein cholesterol, mean hemoglobin A_1c_ level, estimated glomerular filtration rate, and duration of diabetes. Model 3 includes model 2 plus further adjustment for mean BMI.

#### ILI Group

In the ILI group, the HRs per 1-SD increase in the CV of BMI were 1.23 (95% CI, 1.01-1.50) for all-cause mortality, 1.54 (95% CI, 1.05-2.25) for CVD mortality, and 1.06 (95% CI, 0.86-1.31) for CVD events. In quartile analyses, the HRs for quartile 4 (vs quartile 1) of CV of BMI were 0.99 (95% CI, 0.45-2.16), 1.14 (95% CI, 0.12-10.53), and 0.77 (95% CI, 0.40-1.49) for all-cause mortality, CVD-related mortality, and CVD events, respectively (Table 2). Comparable results were observed in both trial groups when variability of BMI was defined using VIM (eTable 3 in the Supplement) or intra-individual SD (eTable 4 in the Supplement).

### Variability of WC and Cardiovascular Outcomes

#### DSE Group

Table 3 displays the HRs by CV of WC. Among participants in the DSE groups, the HRs for each 2.6% (ie, 1-SD) increment in the CV of WC were 1.23 (95% CI, 1.01-1.49) for all-cause mortality, 1.52 (95% CI, 0.96-2.39) for CVD mortality, and 1.08 (95% CI, 0.88-1.33) for CVD events. When analyzed as quartiles, those in quartile 4 had statistically significant higher risks of all-cause mortality (HR, 1.84 [95% CI, 1.01-3.35]) and CVD mortality (HR, 6.46 [95% CI, 1.16-36.01]) but not CVD events (HR, 1.28 [95% CI, 0.72-2.29]) compared with participants in quartile 1.

**Table 3.** Hazard Ratios for Study Outcomes by Coefficient of Variation of Waist Circumference Stratified by Treatment Group in the Action for Health in Diabetes Study

Outcome	Quartiles of coefficient of variation of waist circumference				*P *for trend	Per 2.6% increase
	1, <1.91%	2, 1.91%-2.94%	2, 2.95%-4.49%	4, >4.49%		
**Diabetes support and education group (n = 1775)**						
All-cause mortality						
No. of events/person-years	28/4049.9	21/3338.2	18/2557.6	20/1762.5	NA	87/11 708.2
Rate 1000 person-years (95% CI)	6.9 (4.8-10.0)	6.3 (4.1-9.6)	7.0 (4.4-11.2)	11.3 (7.3-17.6)	NA	7.4 (6.0-9.2)
Hazard ratio (95% CI)^a^						
Model 1	1 [Reference]	0.98 (0.56-1.72)	1.09 (0.60-1.97)	1.89 (1.06-3.36)	.06	1.24 (1.03-1.50)
Model 2	1 [Reference]	0.99 (0.56-1.75)	1.06 (0.58-1.93)	1.79 (0.99-3.26)	.11	1.23 (1.01-1.51)
Model 3	1 [Reference]	1.00 (0.57-1.77)	1.12 (0.62-2.05)	1.84 (1.01-3.35)	.08	1.23 (1.01-1.49)
Cardiovascular death						
No. of events/person-years	2/4049.7	4/3338.2	3/2557.6	5/1761.8	NA	14/11 707.2
Rate 1000 person-years (95% CI)	0.5 (0.1-2.0)	1.2 (0.4-3.2)	1.2 (0.4-3.6)	2.8 (1.2-6.8)	NA	1.2 (0.7-2.0
Hazard ratio (95% CI)^a^						
Model 1	1 [Reference]	2.56 (0.47-13.96)	2.67 (0.44-16.04)	6.76 (1.30-35.14)	.02	1.29 (0.91-1.83)
Model 2	1 [Reference]	2.28 (0.41-12.64)	2.40 (0.39-14.65)	6.14 (1.10-34.08)	.04	1.51 (0.96-2.37)
Model 3	1 [Reference]	2.26 (0.41-12.56)	2.27 (0.37-13.96)	6.46 (1.16-36.01)	.04	1.52 (0.96-2.39)
Cardiovascular events						
No. of events/person-years	39/3921.8	34/3249.0	20/2505.9	19/1705.3	NA	112/11 381.9
Rate per 1000 person-years (95% CI)	9.9 (7.3-13.6)	10.5 (7.5-14.6)	8.0 (5.1-12.4)	11.1 (7.1-17.5)	NA	9.8 (8.2-11.8)
Hazard ratio (95% CI)^a^						
Model 1	1 [Reference]	1.09 (0.69-1.73)	0.85 (0.50-1.47)	1.24 (0.72-2.16)	.75	1.05 (0.85-1.30)
Model 2	1 [Reference]	1.12 (0.70-1.78)	0.94 (0.54-1.62)	1.28 (0.72-2.29)	.61	1.08 (0.88-1.33)
Model 3	1 [Reference]	1.12 (0.70-1.78)	0.94 (0.54-1.62)	1.28 (0.72-2.29)	.61	1.08 (0.88-1.33)
**Intensive lifestyle intervention group (n = 1829)**						
All-cause mortality						
No. of events/person-years	13/1870.4	19/2622.7	12/3335.8	35/4198.5	NA	79/12 027.5
Rate per 1000 person-years (95% CI)	7.0 (4.0-12.0)	7.2 (4.6-11.4)	3.6 (2.0-6.3)	8.3 (6.0-11.6)	NA	6.6 (5.3-8.2)
Hazard ratio (95% CI)^a^						
Model 1	1 [Reference]	1.04 (0.51-2.10)	0.47 (0.22-1.04)	1.09 (0.58-2.07)	.93	1.08 (0.90-1.30)
Model 2	1 [Reference]	0.98 (0.47-2.06)	0.51 (0.22-1.17)	1.18 (0.59-2.34)	.71	1.12 (0.93-1.35)
Model 3	1 [Reference]	0.97 (0.46-2.03)	0.53 (0.23-1.20)	1.23 (0.61-2.46)	.60	1.12 (0.94-1.34)
Cardiovascular death						
No. of events/person-years	4/1870.4	4/2622.7	1/3335.8	10/4198.2	NA	19/12 027.2
Rate per 1000 person-years (95% CI)	2.1 (0.8-5.7)	1.5 (0.6-4.1)	0.3 (0.0-2.1)	2.4 (1.3-4.4)	NA	1.6 (1.0-2.5)
Hazard ratio (95% CI)^a^						
Model 1	1 [Reference]	0.70 (0.17-2.80)	0.12 (0.01-1.12)	0.94 (0.29-3.02)	.99	1.28 (0.96-1.71)
Model 2	1 [Reference]	0.45 (0.10-2.11)	0.11 (0.01-1.02)	0.55 (0.15-2.07)	.43	1.27 (0.90-1.79)
Model 3	1 [Reference]	0.45 (0.10-2.12)	0.11 (0.01-1.03)	0.55 (0.15-2.11)	.44	1.27 (0.90-1.79)
Cardiovascular events						
No. of events/person-years	24/1820.4	18/2590.0	29/3261.9	33/4119.8	NA	104/11 792.1
Rate per 1000 person-years (95% CI)	13.2 (8.8-19.7)	6.9 (4.4-11.0)	8.9 (6.2-12.8)	8.0 (5.7-11.3)	NA	8.8 (7.3-10.7)
Hazard ratio (95% CI)^a^						
Model 1	1 [Reference]	0.53 (0.29-0.99)	0.64 (0.37-1.10)	0.57 (0.33-0.96)	.11	0.98 (0.82-1.18)
Model 2	1 [Reference]	0.43 (0.22-0.85)	0.76 (0.43-1.34)	0.67 (0.38-1.18)	.52	1.08 (0.90-1.28)
Model 3	1 [Reference]	0.43 (0.22-0.83)	0.78 (0.44-1.38)	0.70 (0.39-1.25)	.64	1.08 (0.91-1.28)

Abbreviation: NA, not applicable.

^a^ Model 1 adjusted for age, sex, ,race and ethnicity. Model 2 includes variables in model 1 with further adjustment for current smoking, alcohol drinking, use of antihypertensive medications, mean systolic blood pressure, mean ratio of total to high-density lipoprotein cholesterol, mean hemoglobin A_1c_ level, estimated glomerular filtration rate, and duration of diabetes. Model 3 includes model 2 plus further adjustment for mean waist circumference.

#### ILI Group

Among participants in the ILI group, the HRs for each 2.6% (ie, 1-SD) increase in CV of WC were 1.12 (95% CI, 0.94-1.34) for all-cause mortality, 1.27 (95% CI, 0.90-1.79) for CVD mortality, and 1.08 (95% CI, 0.91-1.28) for CVD events. The HRs for quartile 4 (vs quartile 1) of CV of WC were 1.23 (95% CI, 0.61-2.46) for all-cause mortality, 0.55 (95% CI, 0.15-2.11) for CVD mortality, and 0.70 (95% CI, 0.39-1.25) for CVD events (Table 3). Comparable results were found in both groups when variability in WC was measured using VIM or SD (eTables 5 and 6 in the Supplement).

### Sensitivity Analyses

In supplementary analyses, we evaluated the variability of body weight with outcomes. Consistent with our main analyses, the highest quartile of body weight variability (vs quartile 1) was associated with greater hazards of all-cause deaths (HR, 4.12 [95% CI, 2.21-7.68]), CVD deaths (HR, 15.32 [95% CI, 2.83-82.91]), and CVD events (HR, 2.20 [95% CI, 1.23-3.94]) among participants in the DSE group but not in the ILI group (eTables 7, 8, and 9 in the Supplement). In analyses restricted to individuals for whom weight change between the baseline and fourth visits was less than 5 kg and adjusting for BMI change (or WC change) between the baseline and fourth visits, variability of BMI and WC remained positively associated with higher risks of mortality and CVD events (eTables 10, 11, 12 and 13 in the Supplement).

## Discussion

We evaluated the associations of long-term variability in adiposity indices with clinical outcomes in a large sample of adults with type 2 diabetes enrolled in a weight loss intervention trial: the Look AHEAD study. Among participants assigned to the ILI group, we found no association of the variability in adiposity indices with outcomes, while a positive and consistent association was found between the variability in adiposity indices and CVD outcomes and deaths in the DSE groups.

Our findings imply that weight fluctuations during weight loss attempts among individuals with type 2 diabetes may lead to deleterious health outcomes. While a few studies have investigated the association of body weight variability with CVD outcomes among patients with type 2 diabetes, to our knowledge none of these studies assessed how weight loss intervention affects this association.^8,9,10,19^ The positive association between body weight variability and CVD and mortality among individuals with type 2 diabetes is consistent with the findings of prior studies, albeit limited in number.^8,9,10,19^ It is worthwhile mentioning that, to our knowledge, our study is the first to examine and observe the association between long-term variability of WC—a measure of central obesity—and CVD outcomes and deaths. Our investigation of the variability of WC complements extant literature, as it is well established that BMI does not adequately capture the distribution of body fat.^20^

The exact mechanisms explaining the association between increased variability of obesity measures and adverse outcomes among individuals with type 2 diabetes are incompletely understood, but a few hypotheses have been formulated. Evidence suggests that following initial weight loss (especially from dieting), weight gain results in rapid expansion and hyperplasia of adipose tissue as a result of metabolic shifts that tend to favor lipid accumulation.^21^ Metabolically active adipose tissue then produces an array of adipokines (leptin and others) leading to adverse outcomes. Another possible mechanism involves low-grade inflammation that may occur in those with higher variation in adiposity indices; indeed, body weight fluctuation has been noted to be associated with elevated concentrations of circulating C-reactive protein.^22^ Additionally, body weight fluctuations have been associated with the development of all the components of the metabolic syndrome.^23,24^ Animal studies suggest that weight-cycled mice exhibited a high number of CD4^+^ and CD8^+^ T cells in their adipose tissue with increased production of cytokines, which likely contribute to metabolic dysfunction and adverse outcomes associated with weight cycling.^25^ Body weight variability may affect body fat distribution. Indeed, studies suggest that repeated bouts of weight loss (from dieting) followed by regain may preferentially promote abdominal adiposity, which is associated with adverse CVD outcomes.^21,26^ We found that an intensive weight loss program attenuated the associations of body weight variability with adverse CVD outcomes. While the exact mechanisms for this finding are unclear, a possible explanation relates to the effect of exercise on abdominal fat. Body weight variability achieved through dieting (in contrast to exercise) results in abdominal fat expansion,^21,26^ which likely mediated the increased risk of CVD outcomes in the DSE group of our study. On the other hand, body weight variability associated with higher intensity exercise (in the ILI group) would be expected to be associated with a reduction in abdominal adiposity; indeed, exercise training has been shown to be very effective at reducing total abdominal and visceral fat.^27,28^

In our study, the observed associations were stronger when adiposity was evaluated using BMI compared with WC, suggesting that body weight fluctuations may have a stronger effect on BMI than WC. This may because BMI measures not only fat mass but lean mass, including muscle mass.^29^ The peripheral muscle is a metabolic organ and thus has an influence on outcomes. Lifestyle changes are likely to affect muscular mass, hence BMI and outcomes.^29^

The research and public health implications of our study are manifold. Our analyses suggest that weight loss intervention attenuates the association of body weight (as well as WC) variability with CVD outcomes and mortality. While it is premature to make any specific recommendation for change in clinical practice based on our study, our findings suggest that strategies that involve ILI, including exercise, and those geared toward maintaining weight loss and preventing weight regain should constitute a major focus of weight loss prescriptions for individuals with overweight or obesity and type 2 diabetes. It is worthwhile mentioning that the main Look AHEAD trial analysis showed no beneficial effects of ILI, an intensive weight loss program, on CVD outcomes.^15^

This study has several strengths. First, unlike prior studies of which we are aware, we studied the variability of both overall and abdominal obesity (as measured by WC). Second, we used a large, diverse community-based sample of individuals with type 2 diabetes, in which adiposity indices were measured at regular preset intervals over a 36-month time frame. Finally, our study encompassed a long duration of follow-up, a standardized evaluation of obesity measures, and a blinded ascertainment of outcomes.

### Limitations

Our study should be interpreted in the context of a few limitations. First, given that our sample was limited to people with type 2 diabetes who could complete exercise testing, were motivated to participate in the trial, and could be included in the analysis, the results may not be generalizable to all patients with type 2 diabetes. Second, our study relied on only 4 time points to measure variability of obesity indices; it is therefore possible that we may have underestimated variability, as suggested by data from studies of BP variability.^30^ Third, the number of events was low for certain outcomes (for example CVD deaths); thus, we may have lacked statistical power for these outcomes, especially in our assessment of statistical interaction. Furthermore, our analysis was observational, thus the possibility of residual confounding exists.

## Conclusions

In this study with a large cohort of individuals with type 2 diabetes, an ILI changed the association of long-term variability of adiposity indices with cardiovascular outcomes and deaths. Among individuals who underwent intensive weight loss, a higher variability of adiposity measures was not associated with higher risks of adverse outcomes; whereas, among those in the standard care group, a greater variability of obesity indices was independently associated with elevated risks of all-cause mortality, cardiovascular mortality, and cardiovascular events.

## Notes

Supplement. **eFigure.** Study Design **eMethods. **Ascertainment of Study Outcomes **eTable 1.** Comparison of Baseline Characteristics of Included Study Participants With Those Excluded From the Analytical Sample **eTable 2. **Characteristics of Study Participants by Quartiles of Coefficient of Variation of WC Stratified by Intervention Group in the Look AHEAD Study **eTable 3. **Hazard Ratios for Study Outcomes by Quartiles of VIM of BMI Stratified by Intervention Group in the Look AHEAD Study **eTable 4. **Hazard Ratios for Study Outcomes by Quartiles of SD of BMI Stratified by Intervention Group in the Look AHEAD Study **eTable 5. **Hazard Ratios for Study Outcomes by Quartiles of VIM of WC Stratified by Intervention Group in the Look AHEAD Study **eTable 6. **Hazard Ratios for Study Outcomes by Quartiles of SD of WC Stratified by Intervention Group in the Look AHEAD Study **eTable 7. **Hazard Ratios for Study Outcomes by Quartiles of Coefficient of Variation of Body Weight Stratified by Intervention Group in the Look AHEAD Study **eTable 8. **Hazard Ratios for Study Outcomes by Quartiles of VIM of Body Weight Stratified by Intervention Group in the Look AHEAD Study **eTable 9. **Hazard Ratios for Study Outcomes by Quartiles of SD of Body Weight Stratified by Intervention Group in the Look AHEAD Study **eTable 10. **Hazard Ratios for Study Outcomes by Coefficient of Variation of BMI and Treatment Group After Additional Adjustment for BMI Change **eTable 11. **Hazard Ratios for Study Outcomes by Coefficient of Variation of WC and Treatment Group After Additional Adjustment for WC Change **eTable 12. **Hazard Ratios for Study Outcomes by Coefficient of Variation of BMI and Treatment Group After Restricting Analyses to Participants With Weight Change Less Than 5 kg **eTable 13. **Hazard Ratios for Study Outcomes by Coefficient of Variation of Waist Circumference and Treatment Group After Restricting Analyses to Participants With Weight Change Less Than 5 kg
