# Supplementary material for: Body Weight Variability and Risk of Cardiovascular Outcomes and Death in the Context of Weight Loss Intervention Among Patients With Type 2 Diabetes
Source: JAMA Netw Open. 2022 Feb 18;5(2):e220055. doi: 10.1001/jamanetworkopen.2022.0055 (PMC8857684; doi:10.1001/jamanetworkopen.2022.0055)
Supplement: Supplement. — eFigure. Study Design eMethods. Ascertainment of Study Outcomes eTable 1. Comparison of Baseline Characteristics of Included Study Participants With Those Excluded From the Analytical Sample eTable 2. Characteristics of Study Participants by Quartiles of Coefficient of Variation of WC Stratified by Intervention Group in the Look AHEAD Study eTable 3. Hazard Ratios for Study Outcomes by Quartiles of VIM of BMI Stratified by Intervention Group in the Look AHEAD Study eTable 4. Hazard Ratios for Study Outcomes by Quartiles of SD of BMI Stratified by Intervention Group in the Look AHEAD Study eTable 5. Hazard Ratios for Study Outcomes by Quartiles of VIM of WC Stratified by Intervention Group in the Look AHEAD Study eTable 6. Hazard Ratios for Study Outcomes by Quartiles of SD of WC Stratified by Intervention Group in the Look AHEAD Study eTable 7. Hazard Ratios for Study Outcomes by Quartiles of Coefficient of Variation of Body Weight Stratified by Intervention Group in the Look AHEAD Study eTable 8. Hazard Ratios for Study Outcomes by Quartiles of VIM of Body Weight Stratified by Intervention Group in the Look AHEAD Study eTable 9. Hazard Ratios for Study Outcomes by Quartiles of SD of Body Weight Stratified by Intervention Group in the Look AHEAD Study eTable 10. Hazard Ratios for Study Outcomes by Coefficient of Variation of BMI and Treatment Group After Additional Adjustment for BMI Change eTable 11. Hazard Ratios for Study Outcomes by Coefficient of Variation of WC and Treatment Group After Additional Adjustment for WC Change eTable 12. Hazard Ratios for Study Outcomes by Coefficient of Variation of BMI and Treatment Group After Restricting Analyses to Participants With Weight Change Less Than 5 kg eTable 13. Hazard Ratios for Study Outcomes by Coefficient of Variation of Waist Circumference and Treatment Group After Restricting Analyses to Participants With Weight Change Less Than 5 kg [file jamanetwopen-e220055-s001.pdf]

Supplemental Online Content

Kaze AD, Santhanam P, Erqou S, Ahima RS, Bertoni AG, Echouffo-Tcheugui JB. Body weight variability and risk of cardiovascular outcomes and death in the context of weight loss intervention among patients with type 2 diabetes. *JAMA Netw Open.* 2022;5(2):e220055. doi:10.1001/jamanetworkopen.2022.0055

eFigure. Study Design

eMethods. Ascertainment of Study Outcomes

eTable 1. Comparison of Baseline Characteristics of Included Study Participants With Those Excluded From the Analytical Sample

eTable 2. Characteristics of Study Participants by Quartiles of Coefficient of Variation of WC Stratified by Intervention Group in the Look AHEAD Study

eTable 3. Hazard Ratios for Study Outcomes by Quartiles of VIM of BMI Stratified by Intervention Group in the Look AHEAD Study

eTable 4. Hazard Ratios for Study Outcomes by Quartiles of SD of BMI Stratified by Intervention Group in the Look AHEAD Study

eTable 5. Hazard Ratios for Study Outcomes by Quartiles of VIM of WC Stratified by Intervention Group in the Look AHEAD Study

eTable 6. Hazard Ratios for Study Outcomes by Quartiles of SD of WC Stratified by Intervention Group in the Look AHEAD Study

eTable 7. Hazard Ratios for Study Outcomes by Quartiles of Coefficient of Variation of Body Weight Stratified by Intervention Group in the Look AHEAD Study

eTable 8. Hazard Ratios for Study Outcomes by Quartiles of VIM of Body Weight Stratified by Intervention Group in the Look AHEAD Study

eTable 9. Hazard Ratios for Study Outcomes by Quartiles of SD of Body Weight Stratified by Intervention Group in the Look AHEAD Study

eTable 10. Hazard Ratios for Study Outcomes by Coefficient of Variation of BMI and Treatment Group After Additional Adjustment for BMI Change

eTable 11. Hazard Ratios for Study Outcomes by Coefficient of Variation of WC and Treatment Group After Additional Adjustment for WC Change

eTable 12. Hazard Ratios for Study Outcomes by Coefficient of Variation of BMI and Treatment Group After Restricting Analyses to Participants With Weight Change Less Than 5 kg

eTable 13. Hazard Ratios for Study Outcomes by Coefficient of Variation of Waist Circumference and Treatment Group After Restricting Analyses to Participants With Weight Change Less Than 5 kg

This supplemental material has been provided by the authors to give readers additional information about their work.

© 2022 Kaze AD et al. *JAMA Network Open.*

eFigure. Study Design

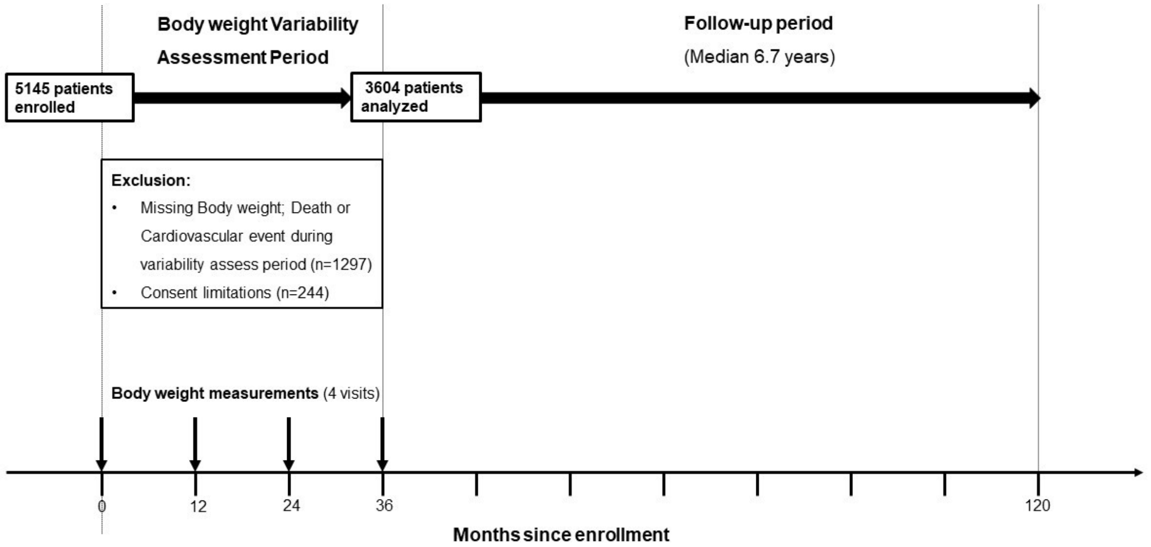

Figure S1

© 2022 Kaze AD et al. *JAMA Network Open.*

eMethods. Ascertainment of Study Outcomes

Adjudication of Cardiovascular events

Cardiovascular event included cardiovascular deaths (including fatal myocardial infarctions [MI] and strokes, and other cardiovascular causes), non-fatal myocardial infarctions, non-fatal strokes.

Myocardial Infarction

The algorithm for classifying MI included elements of the history, results of cardiac enzyme determinations, and ECG readings, and included MI that occurred during surgery/procedure and MI aborted by thrombolytic therapy or procedure. The definition and differentiation of definite vs probable vs possible MI followed published consensus criteria on case definitions of acute coronary heart disease, with classification at the highest level of the combinations of the three characteristics. Cardiac symptoms were defined as presence of acute chest pain, epigastric, neck jaw, or arm pain or discomfort or pressure without apparent noncardiac source. More general, atypical symptoms, such as fatigue, nausea, vomiting, diaphoresis, faintness, and back pain, were not used as diagnostic criterion. Cardiac signs included acute congestive heart failure or cardiogenic shock in the absence of non-CHD cause.

The adjudicators interpreted serial tracings, with the ECG series assigned the highest category for which criteria were met. Evolving diagnostic included new major Q wave in the absence of Q wave in previous ECG, or the presence of an equivocal Q wave in previous ECG followed by the appearance of major Q wave and either major ST-segment depression, T wave inversion, or ST-segment elevation. Positive ECG included evolving ST elevation alone, evolving equivocal Q wave plus evolving ST-T depression/inversion, or new left bundle block.

Nonspecific ECG included evolving non-ST elevation non-Q wave pattern (eg ST depression

© 2022 Kaze AD et al. *JAMA Network Open.*

alone) or evolving minor Q wave alone. Findings other than these or normal ECGs were classified as negative for ischemia. Look AHEAD clinical centers provided copies of the 12-lead ECG obtained at study visits in the adjudication packet.

Biomarkers were interpreted in the clinical context, with evaluation of pattern and timing. An adequate set of biomarkers was defined as at least two measurements of the same marker taken at least 6 hours apart. The general classification pertained to cases without defibrillation/cardioversion, CPR, or intervention. Diagnostic biomarkers were defined as at least 1 positive biomarker in an adequate set showing a rising or falling pattern in the setting of clinical cardiac ischemia and the absence of noncardiac (absence of overt ischemic heart disease) causes of biomarker elevation. Positive biomarkers were defined as at least one value of 2 or more times the upper limit of normal in the lab performing the measurement, as described below. Equivocal biomarkers were defined as only 1 available measurement that was positive, or a rising or falling pattern not in the setting of clinical cardiac ischemia or in the presence of nonischemic causes of biomarker elevation. Equivocal also included the range between "above normal" and "twice the upper limit of normal. Normal biomarkers did not meet criteria for positive or equivocal biomarkers. In the first 48 hours following percutaneous angioplasty, levels of CK or MB or troponin above 3 times the upper limit of normal were characterized as positive. Troponin took precedence over CK-MB, and CK-MB took precedence over CK if both were available. Similarly for coronary artery bypass graft surgery, levels of troponin or MB

© 2022 Kaze AD et al. *JAMA Network Open.*

above 5 times the upper limit of normal within 48 hours of the procedure were categorized as positive. The consensus criteria defined "positive" as at least one value exceeding the 99th percentile of the distribution in healthy populations or the lowest level at which a 10% coefficient of variation can be demonstrated for that value. Additional literature described an array of assay specific values for the cut-off point of the 99th percentile. In practice, the adjudication committee could not operationally define these cut-off points due to variation in the assays used by clinical labs and the lack of available information on these ranges specific to the large number of clinical labs performing biomarkers on Look AHEAD participants. The committee continued to use the cut-off point of 2 times the upper limit of normal for positive, and the range between less than 2 times the upper limit of normal and the upper limit of normal for equivocal, since these levels closely corresponded to the 99th percentile where that could be ascertained.

Stroke

The minimum criterion for definite or probable stroke is evidence of sudden or rapid onset of focal neurological symptoms lasting more than 24 hours or leading to death, in the absence of evidence for a non-stroke cause. Exclusionary conditions for stroke included major brain trauma, intracranial neoplasm, coma due to metabolic disorders or disorders of fluid or electrolyte balance, peripheral neuropathy, or central nervous system infections. Major stroke symptoms were hemiparesis of two or more body parts, homonymous hemianopia, or aphasia.

Minor stroke symptoms were diplopia, vertigo and gait disturbance (both together), dysarthria,

© 2022 Kaze AD et al. *JAMA Network Open.*

dysphagia, dysphonia, or unilateral numbness of one or more body parts.

Cardiovascular Death

Coronary heart disease deaths were classified using the case definitions for acute CHD, separating into inpatient and out of hospital deaths and classified into hierarchical order. CHD death occurring in the hospital included definite fatal MI, probable fatal MI, and possible fatal coronary event. Definite fatal MI was defined as death within 28 days of hospital admission in MI cases, defined as definite MI above; or postmortem findings consistent with MI within 28 days. Probable fatal MI was defined as death within 28 days of hospital admission in MI cases defined as probable MI above; or death within 6 hours of hospital admission with cardiac symptoms and/or signs and other confirmatory information (biomarkers, ECG) were absent or not diagnostic. Possible fatal coronary event was defined as death within 28 days of hospital admission in case defined as possible MI or angina; or postmortem findings show old infarct and/or > 50% atherosclerotic narrowing of coronary arteries. Out of hospital CHD deaths included definite fatal MI, definite fatal CHD, and possible fatal CHD. Definite fatal MI (outpatient) was defined as documented definite or probable MI in the previous 28 days and no evidence of a non-coronary cause of death; or autopsy evidence of recent coronary occlusion or MI less than 28 days old. Definite fatal CHD was defined as a history of CHD and/or documented cardiac pain within 72 hours before death; and no evidence of a non-coronary cause of death; or autopsy evidence of chronic CHD, including coronary atherosclerosis and

myocardial scarring. Possible fatal CHD was defined as an ICD code for underlying cause of death (ICD 9 410 to 414, 427.5, 429.2, and 799; ICD 10 I20 to 25 and 146) and no evidence of a non-coronary cause of death.

Look AHEAD clinical staff blinded to randomization assignment were trained to conduct a structured interview with a knowledgeable informant to collect data on circumstances, symptoms, and other information useful for determining cause of death in cases of outpatient death (including participants pronounced death in the emergency department) not obviously due to a non-CHD cause. Informant interviews were available for adjudicators, who also had access to the records from the most recent prior hospitalization, and a summary report of all prior outcomes cases reported, and the adjudicated outcome, during the course of the study.

Cardiovascular disease (CVD) deaths also included deaths due to congestive heart failure, documented arrhythmia, stroke, and other CVD. Documented arrhythmia death was defined as death due to bradyarrhythmias or tachyarrhythmias not associated with an acute cardiac ischemic event. Stroke death was defined as death due to stroke, any subtype, occurring within seven days of the signs and symptoms of a stroke and/or prior to hospital discharge. Other CVD death was defined as death due to other vascular diseases including abdominal aortic aneurysm rupture.

© 2022 Kaze AD et al. *JAMA Network Open.*

eTable 1. Comparison of Baseline Characteristics of Included Study Participants With Those Excluded From the Analytical Sample

Characteristic	Excluded (n = 1,302)	Included (n = 3,604)	P value
Age, mean (SD), years	60.3 (6.8)	58.4 (6.7)	<0.001
Sex			<0.001
Women, %	48.7	62.2	
Men, %	51.3	37.8	
Race/ethnicity, %			<0.001
Black	13.5	17.4	
Hispanic	17.3	12.5	
Other*	4.1	3.4	
White	65.1	66.7	
Current smoking, %	5.9	3.6	<0.001
Alcohol drinking, %	32.0	33.5	0.33
Diabetes duration, median (IQR), years	6.0 (2.0-11.0)	5.0 (2.0-9.0)	<0.001
eGFR, mean (SD), mL/min/1.73m^2	87.7 (16.9)	90.9 (15.7)	<0.001
Hemoglobin A_{1C}, mean (SD), %	7.4 (1.2)	7.2 (1.1)	<0.001
Total-to-HDL cholesterol ratio, mean (SD)	4.7 (1.4)	4.7 (1.5)	0.12
Total cholesterol, mean (SD), mg/dL	188.2 (38.8)	192.4 (36.9)	<0.001
HDL cholesterol, mean (SD), mg/dL	42.1 (11.5)	44.0 (12.0)	<0.001
LDL cholesterol, mean (SD), mg/dL	109.5 (32.8)	113.7 (32.0)	<0.001
Use of BP-lowering drug, %	81.1	71.3	<0.001
Systolic BP, mean (SD), mm Hg	129.6 (17.6)	128.9 (16.9)	0.24
Diastolic BP, mean (SD), mm Hg	70.0 (9.7)	70.3 (9.5)	0.36
BMI, mean (SD)	35.7 (5.6)	36.0 (5.9)	0.09
WC, mean (SD), cm	115.1 (13.7)	113.5 (14.1)	<0.001

AHEAD indicates Action for Health in Diabetes; BMI, body mass index (calculated as weight in kilograms divided by height in meters squared); BP, blood pressure; eGFR, estimated glomerular filtration rate; HDL, high-density lipoprotein; IQR, interquartile range; LDL, low-density lipoprotein; SD, standard deviation; WC, waist circumference.

* Race and ethnicity information were self-reported by participants. Other race and ethnicity includes race and ethnicity indicated specifically as Asian or Pacific Islander, North American Native, biracial, other, or unknown.

© 2022 Kaze AD et al. *JAMA Network Open.*

eTable 2. Characteristics of Study Participants by Quartiles of Coefficient of Variation of WC Stratified by Intervention Group in the Look AHEAD Study

	Diabetes Support and Education (n = 1775)						Intensive Lifestyle Intervention (n = 1829)				
	Quartiles of Coefficient of Variation of WC, %						Quartiles of Coefficient of Variation of WC, %				
	< 1.91 (n = 616)	1.91-2.94 (n = 504)	2.95-4.49 (n = 389)	> 4.49 (n = 266)	*P* value		< 1.91 (n = 291)	1.91-2.94 (n = 395)	2.95-4.49 (n = 510)	> 4.49 (n = 633)	*P* value
**At Baseline**											
Age, mean (SD), years	58.9 (6.7)	58.6 (6.5)	58.7 (6.8)	57.8 (7.1)	0.19		57.7 (6.4)	57.8 (6.6)	58.6 (6.5)	58.5 (6.7)	0.09
Sex, %					<0.001						0.02
Men	43.9	38.7	33.6	29.3			37.1	33.4	36.5	42.2	
Women	56.0	61.3	65.8	71.1			63.0	66.8	63.1	57.5	
Race/ethnicity, %					0.96						0.10
Black	17.5	15.9	19.0	17.3			16.9	22.3	15.9	15.0	
Hispanic	11.5	12.1	13.1	12.8			14.4	12.4	12.9	11.4	
Other	3.3	3.2	3.7	2.7			3.9	2.8	4.3	3.0	
White	67.7	68.9	64.3	67.3			64.8	62.5	66.7	70.5	
Current smoking, %	3.1	4.0	2.1	3.8	0.41		4.9	5.1	2.8	4.0	0.27
Alcohol drinking, %	36.7	35.1	29.6	28.6	0.03		37.0	30.1	33.9	34.6	0.28
Diabetes duration, median (IQR) years	5.0 (2.0-9.0)	5.0 (2.0-9.0)	5.0 (2.0-9.0)	5.0 (2.0-8.0)	0.98		5.0 (2.0-9.5)	5.0 (2.0-9.0)	5.0 (2.0-9.0)	5.0 (2.0-10.0)	0.92
eGFR, mean (SD), mL/min/1.73m^2	90.5 (14.9)	90.7 (15.1)	90.8 (15.9)	91.4 (17.3)	0.89		92.5 (15.7)	92.7 (16.0)	90.1 (16.2)	89.8 (15.3)	0.006
**During Variability Assessment Period**											
Mean hemoglobin A_{1C}, mean (SD), %	7.1 (1.0)	7.2 (1.0)	7.1 (1.0)	7.0 (1.0)	0.20		7.2 (1.1)	7.2 (1.1)	6.8 (0.9)	6.6 (0.9)	<0.001
Mean total-to-HDL cholesterol ratio, mean (SD)	4.4 (1.2)	4.4 (1.3)	4.1 (1.1)	4.1 (1.1)	<0.001		4.4 (1.2)	4.3 (1.2)	4.2 (1.2)	4.0 (1.1)	<0.001
Use of BP-lowering drug, %	84.3	85.4	83.6	82.4	0.73		81.9	82.9	82.2	82.3	0.99
Mean systolic BP, mean (SD), mm Hg	127.7 (13.7)	127.3 (13.6)	127.5 (12.9)	125.0 (13.4)	0.04		126.2 (13.9)	126.1 (14.4)	122.9 (13.7)	122.7 (14.4)	<0.001
Mean diastolic BP, mean (SD), mm Hg	69.2 (8.0)	69.1 (8.1)	68.7 (7.4)	67.3 (7.9)	0.01		69.0 (8.2)	68.9 (8.4)	67.6 (8.0)	67.5 (7.8)	0.006
Mean BMI, mean (SD)	35.8 (5.7)	36.0 (5.7)	36.0 (5.6)	35.5 (6.2)	0.71		35.1 (6.0)	36.0 (6.3)	33.8 (5.8)	32.4 (5.3)	<0.001
Mean WC, mean (SD), cm	113.8 (13.4)	113.3 (13.2)	112.5 (12.6)	110.8 (13.2)	0.02		112.3 (13.9)	112.7 (13.8)	108.1 (13.5)	105.4 (13.0)	<0.001

Data are mean (SD), median (interquartile range), or proportion as appropriate.

AHEAD indicates Action for Health in Diabetes; eGFR, estimated glomerular filtration rate; HDL, high-density lipoprotein; BMI, body mass index (calculated as weight in kilograms divided by height in meters squared); WC, waist circumference; BP, blood pressure; IQR, interquartile range; SD, standard deviation.

© 2022 Kaze AD et al. *JAMA Network Open.*

eTable 3. Hazard Ratios for Study Outcomes by Quartiles of VIM of BMI Stratified by Intervention Group in the Look AHEAD Study

	Quartiles of VIM of Body Mass Index				P_{trend}	Per 14.5-unit
	< 9.59	9.59-15.33	15.33-23.90	> 23.90		
**Diabetes Support and Education (n = 1775)**						
**All-cause mortality**						
Model 1	Reference	1.46 (0.73-2.91)	1.71 (0.86-3.40)	3.12 (1.66-5.87)	<0.001	1.44 (1.23-1.68)
Model 2	Reference	1.39 (0.70-2.79)	1.67 (0.84-3.32)	3.07 (1.63-5.78)	<0.001	1.45 (1.23-1.70)
Model 3	Reference	1.39 (0.70-2.79)	1.64 (0.83-3.27)	2.95 (1.56-5.59)	0.001	1.43 (1.21-1.68)
**Cardiovascular death**						
Model 1	Reference	0.75 (0.07-8.29)	5.82 (1.13-30.15)	9.71 (1.85-50.98)	0.004	1.54 (1.13-2.10)
Model 2	Reference	0.58 (0.05-6.43)	4.77 (0.90-25.26)	8.12 (1.54-42.95)	0.009	1.60 (1.14-2.24)
Model 3	Reference	0.58 (0.05-6.49)	5.24 (0.98-28.13)	13.25 (2.33-75.27)	0.005	1.73 (1.23-2.44)
**Cardiovascular event**						
Model 1	Reference	1.42 (0.89-2.26)	1.40 (0.83-2.37)	1.64 (0.91-2.94)	0.08	1.11 (0.91-1.34)
Model 2	Reference	1.30 (0.81-2.09)	1.49 (0.87-2.53)	1.69 (0.94-3.04)	0.05	1.15 (0.95-1.39)
Model 3	Reference	1.32 (0.82-2.12)	1.53 (0.90-2.60)	1.80 (0.99-3.27)	0.03	1.17 (0.97-1.42)
**Intensive Lifestyle Intervention (n = 1829)**						
**All-cause mortality**						
Model 1	Reference	1.00 (0.44-2.27)	0.86 (0.39-1.86)	0.95 (0.45-2.03)	0.86	1.10 (0.90-1.34)
Model 2	Reference	0.98 (0.42-2.32)	0.82 (0.36-1.89)	1.15 (0.51-2.58)	0.67	1.21 (0.99-1.46)
Model 3	Reference	0.98 (0.41-2.31)	0.82 (0.36-1.88)	1.14 (0.51-2.58)	0.67	1.21 (0.99-1.47)
**Cardiovascular death**						
Model 1	Reference	2.22 (0.25-19.96)	1.88 (0.22-15.84)	1.88 (0.23-15.43)	0.79	1.27 (0.93-1.74)
Model 2	Reference	1.82 (0.20-16.61)	0.65 (0.06-6.57)	1.35 (0.15-12.39)	0.95	1.46 (1.02-2.09)
Model 3	Reference	1.77 (0.19-16.23)	0.66 (0.07-6.66)	1.31 (0.14-12.21)	0.93	1.49 (1.02-2.19)
**Cardiovascular event**						
Model 1	Reference	0.72 (0.38-1.37)	0.71 (0.39-1.29)	0.59 (0.33-1.07)	0.11	0.93 (0.76-1.15)
Model 2	Reference	0.75 (0.38-1.49)	0.76 (0.39-1.45)	0.75 (0.39-1.45)	0.51	1.05 (0.85-1.29)
Model 3	Reference	0.75 (0.38-1.48)	0.75 (0.39-1.45)	0.75 (0.39-1.45)	0.51	1.05 (0.85-1.29)

Data are hazard ratios (95% confidence intervals) unless otherwise indicated.

Model 1 adjusted for age, sex, race/ethnicity.

Model 2 includes variables in model 1 with further adjustment for current smoking, alcohol drinking, use of antihypertensive medications, average systolic blood pressure, average ratio of total to high-density lipoprotein cholesterol, average hemoglobin A_{1C}, estimated glomerular filtration rate, and duration of diabetes.

Model 3 includes model 2 plus further adjustment for average BMI.

AHEAD indicates Action for Health in Diabetes; BMI, body mass index; VIM, variability independent of the mean.

* P<0.05, † P<0.01, ‡ P<0.001

© 2022 Kaze AD et al. *JAMA Network Open.*

eTable 4. Hazard Ratios for Study Outcomes by Quartiles of SD of BMI Stratified by Intervention Group in the Look AHEAD Study

	Quartiles of SD of BMI, kg/m²				P_{trend}	Per 1.1 kg/m²
	< 0.686	0.686-1.095	1.096-1.704	> 1.705		
**Diabetes Support and Education (n = 1775)**						
**All-cause mortality**						
Model 1	Reference	1.48 (0.84-2.63)	2.00 (1.09-3.66)	3.25 (1.77-5.95)	<0.001	1.41 (1.21-1.64)
Model 2	Reference	1.41 (0.79-2.51)	1.97 (1.07-3.61)	3.13 (1.70-5.78)	<0.001	1.41 (1.21-1.64)
Model 3	Reference	1.40 (0.78-2.49)	1.93 (1.04-3.55)	3.00 (1.58-5.68)	0.001	1.39 (1.18-1.63)
**Cardiovascular death**						
Model 1	Reference	0.76 (0.07-8.46)	6.06 (1.17-31.46)	9.36 (1.77-49.58)	0.001	1.48 (1.10-1.99)
Model 2	Reference	0.58 (0.05-6.50)	5.34 (1.03-27.79)	6.32 (1.16-34.45)	0.006	1.48 (1.07-2.05)
Model 3	Reference	0.63 (0.06-7.03)	6.64 (1.24-35.48)	12.55 (2.07-76.10)	0.001	1.66 (1.19-2.31)
**Cardiovascular event**						
Model 1	Reference	1.59 (1.00-2.52)	1.36 (0.79-2.34)	1.58 (0.88-2.85)	0.12	1.09 (0.90-1.32)
Model 2	Reference	1.46 (0.91-2.33)	1.44 (0.83-2.50)	1.56 (0.86-2.84)	0.11	1.12 (0.93-1.35)
Model 3	Reference	1.49 (0.93-2.39)	1.53 (0.88-2.67)	1.75 (0.94-3.26)	0.05	1.15 (0.95-1.39)
**Intensive Lifestyle Intervention (n = 1829)**						
**All-cause mortality**						
Model 1	Reference	0.92 (0.40-2.12	1.10 (0.51-2.34)	1.06 (0.50-2.26)	0.73	1.08 (0.88-1.34)
Model 2	Reference	1.13 (0.45-2.81	1.23 (0.52-2.89)	1.44 (0.61-3.39)	0.34	1.17 (0.96-1.43)
Model 3	Reference	1.12 (0.45-2.79	1.23 (0.53-2.90)	1.46 (0.62-3.44)	0.32	1.18 (0.97-1.44)
**Cardiovascular death**						
Model 1	Reference	2.40 (0.27-21.57)	2.48 (0.30-20.42)	1.95 (0.23-16.37)	0.78	1.21 (0.86-1.71)
Model 2	Reference	2.07 (0.23-18.95)	1.03 (0.11-9.70)	1.36 (0.14-12.75)	0.88	1.36 (0.92-2.02)
Model 3	Reference	2.01 (0.22-18.50)	1.08 (0.11-10.28)	1.51 (0.16-14.31)	0.99	1.44 (0.97-2.15)
**Cardiovascular event**						
Model 1	Reference	0.86 (0.46-1.61)	0.69 (0.38-1.26)	0.68 (0.38-1.24)	0.16	0.94 (0.76-1.17)
Model 2	Reference	0.82 (0.42-1.59)	0.66 (0.34-1.26)	0.78 (0.41-1.48)	0.43	1.02 (0.82-1.27)
Model 3	Reference	0.81 (0.42-1.58)	0.66 (0.35-1.27)	0.79 (0.41-1.50)	0.47	1.03 (0.83-1.28)

Data are hazard ratios (95% confidence intervals) unless otherwise indicated.

Model 1 adjusted for age, sex, race/ethnicity.

Model 2 includes variables in model 1 with further adjustment for current smoking, alcohol drinking, use of antihypertensive medications, average systolic blood pressure, average ratio of total to high-density lipoprotein cholesterol, average hemoglobin A_{1C}, estimated glomerular filtration rate, and duration of diabetes.

Model 3 includes model 2 plus further adjustment for average BMI.

AHEAD indicates Action for Health in Diabetes; BMI, body mass index; SD standard deviation.

© 2022 Kaze AD et al. *JAMA Network Open.*

eTable 5. Hazard Ratios for Study Outcomes by Quartiles of VIM of WC Stratified by Intervention Group in the Look AHEAD Study

	Quartiles of VIM of Waist Circumference				P_{trend}	Per 237-unit
	< 170.00	170.00-263.02	263.03-394.80	> 394.80		
**Diabetes Support and Education (n = 1775)**						
**All-cause mortality**						
Model 1	Reference	0.87 (0.48-1.58)	1.17 (0.65-2.08)	2.08 (1.18-3.65)	0.02	1.23 (1.04-1.45)
Model 2	Reference	0.85 (0.47-1.56)	1.11 (0.62-1.98)	1.96 (1.10-3.51)	0.04	1.22 (1.02-1.45)
Model 3	Reference	0.82 (0.45-1.49)	1.09 (0.61-1.95)	1.83 (1.02-3.29)	0.06	1.19 (1.00-1.42)
**Cardiovascular death**						
Model 1	Reference	1.90 (0.32-11.35)	2.44 (0.41-14.64)	7.31 (1.47-36.37)	0.01	1.25 (0.93-1.68)
Model 2	Reference	1.67 (0.28-10.18)	2.24 (0.37-13.53)	6.31 (1.21-33.02)	0.02	1.45 (0.95-2.21)
Model 3	Reference	1.70 (0.28-10.42)	2.21 (0.36-13.43)	7.80 (1.44-42.23)	0.02	1.50 (0.99-2.27)
**Cardiovascular event**						
Model 1	Reference	1.07 (0.67-1.71)	0.91 (0.54-1.54)	1.25 (0.73-2.16)	0.65	1.05 (0.86-1.28)
Model 2	Reference	1.09 (0.67-1.75)	0.96 (0.56-1.63)	1.28 (0.72-2.26)	0.58	1.06 (0.88-1.29)
Model 3	Reference	1.09 (0.67-1.76)	0.96 (0.56-1.63)	1.28 (0.72-2.27)	0.58	1.06 (0.88-1.29)
**Intensive Lifestyle Intervention (n = 1829)**						
**All-cause mortality**						
Model 1	Reference	0.89 (0.44-1.77)	0.43 (0.20-0.95)	1.06 (0.57-1.99)	0.85	1.09 (0.91-1.29)
Model 2	Reference	0.97 (0.47-1.98)	0.42 (0.18-0.98)	1.17 (0.60-2.30)	0.78	1.11 (0.94-1.31)
Model 3	Reference	0.96 (0.47-1.96)	0.42 (0.18-0.98)	1.17 (0.60-2.29)	0.77	1.10 (0.93-1.30)
**Cardiovascular death**						
Model 1	Reference	0.31 (0.06-1.72)	0.39 (0.09-1.75)	0.95 (0.29-3.07)	0.59	1.23 (0.94-1.61)
Model 2	Reference	0.33 (0.06-1.86)	0.25 (0.04-1.42)	0.63 (0.17-2.37)	0.66	1.19 (0.87-1.63)
Model 3	Reference	0.33 (0.06-1.86)	0.25 (0.04-1.42)	0.63 (0.17-2.37)	0.66	1.19 (0.86-1.66)
**Cardiovascular event**						
Model 1	Reference	0.70 (0.38-1.29)	0.89 (0.50-1.56)	0.70 (0.40-1.23)	0.38	1.01 (0.85-1.21)
Model 2	Reference	0.82 (0.43-1.56)	0.92 (0.50-1.68)	0.83 (0.45-1.53)	0.67	1.08 (0.91-1.27)
Model 3	Reference	0.82 (0.43-1.55)	0.91 (0.50-1.67)	0.83 (0.45-1.52)	0.67	1.07 (0.91-1.26)

Data are hazard ratios (95% confidence intervals) unless otherwise indicated.
Model 1 adjusted for age, sex, race/ethnicity.
Model 2 includes variables in model 1 with further adjustment for current smoking, alcohol drinking, use of antihypertensive medications, average systolic blood pressure, average ratio of total to high-density lipoprotein cholesterol, average hemoglobin A1C, estimated glomerular filtration rate, and duration of diabetes.
Model 3 includes model 2 plus further adjustment for average waist circumference.
AHEAD indicates Action for Health in Diabetes; VIM, variability independent of the mean.

© 2022 Kaze AD et al. *JAMA Network Open.*

eTable 6. Hazard Ratios for Study Outcomes by Quartiles of SD of WC Stratified by Intervention Group in the Look AHEAD Study

	Quartiles of SD of Waist Circumference, cm				P_{trend}	Per 2.9 cm
	< 2.110	2.110-3.263	3.264-4.896	> 4.897		
**Diabetes Support and Education (n = 1775)**						
**All-cause mortality**						
Model 1	Reference	0.95 (0.53-1.72)	1.15 (0.64-2.08)	2.24 (1.28-3.93)	0.01	1.23 (1.04-1.45)
Model 2	Reference	0.94 (0.52-1.70)	1.09 (0.60-1.97)	2.12 (1.19-3.79)	0.02	1.22 (1.02-1.45)
Model 3	Reference	0.90 (0.49-1.63)	1.06 (0.58-1.93)	1.97 (1.10-3.53)	0.04	1.19 (0.99-1.42)
**Cardiovascular death**						
Model 1	Reference	1.90 (0.32-11.37)	2.44 (0.41-14.62)	7.23 (1.45-36.00)	0.01	1.25 (0.93-1.68)
Model 2	Reference	1.66 (0.27-10.09)	2.26 (0.37-13.70)	6.14 (1.17-32.12)	0.03	1.45 (0.95-2.21)
Model 3	Reference	1.70 (0.28-10.38)	2.22 (0.37-13.51)	7.70 (1.42-41.82)	0.02	1.50 (0.99-2.26)
**Cardiovascular event**						
Model 1	Reference	1.01 (0.63-1.61)	0.88 (0.52-1.49)	1.21 (0.70-2.08)	0.73	1.05 (0.86-1.28)
Model 2	Reference	1.03 (0.64-1.66)	0.93 (0.55-1.58)	1.23 (0.70-2.17)	0.67	1.06 (0.88-1.28)
Model 3	Reference	1.03 (0.64-1.66)	0.93 (0.55-1.58)	1.23 (0.70-2.18)	0.66	1.06 (0.88-1.29)
**Intensive Lifestyle Intervention (n = 1829)**						
**All-cause mortality**						
Model 1	Reference	0.93 (0.47-1.85)	0.39 (0.17-0.89)	1.07 (0.57-2.01)	0.91	1.09 (0.91-1.29)
Model 2	Reference	1.03 (0.51-2.09)	0.37 (0.15-0.90)	1.18 (0.61-2.32)	0.85	1.11 (0.94-1.31)
Model 3	Reference	1.02 (0.50-2.07)	0.37 (0.15-0.90)	1.18 (0.60-2.31)	0.85	1.10 (0.93-1.30)
**Cardiovascular death**						
Model 1	Reference	0.31 (0.06-1.72)	0.39 (0.09-1.76)	0.95 (0.29-3.09)	0.58	1.22 (0.93-1.60)
Model 2	Reference	0.33 (0.06-1.87)	0.25 (0.04-1.42)	0.63 (0.17-2.38)	0.66	1.19 (0.87-1.63)
Model 3	Reference	0.33 (0.06-1.86)	0.25 (0.04-1.42)	0.63 (0.17-2.39)	0.67	1.19 (0.85-1.66)
**Cardiovascular event**						
Model 1	Reference	0.70 (0.38-1.29)	0.89 (0.51-1.56)	0.70 (0.40-1.24)	0.40	1.02 (0.85-1.21)
Model 2	Reference	0.83 (0.44-1.57)	0.92 (0.50-1.67)	0.84 (0.46-1.54)	0.69	1.08 (0.92-1.27)
Model 3	Reference	0.82 (0.43-1.56)	0.91 (0.50-1.66)	0.83 (0.45-1.54)	0.68	1.07 (0.91-1.25)

Data are hazard ratios (95% confidence intervals) unless otherwise indicated.
Model 1 adjusted for age, sex, race/ethnicity.
Model 2 includes variables in model 1 with further adjustment for current smoking, alcohol drinking, use of antihypertensive medications, average systolic blood pressure, average ratio of total to high-density lipoprotein cholesterol, average hemoglobin A_{1C}, estimated glomerular filtration rate, and duration of diabetes.
Model 3 includes model 2 plus further adjustment for average waist circumference.
AHEAD indicates Action for Health in Diabetes; SD, standard deviation.

© 2022 Kaze AD et al. *JAMA Network Open.*

eTable 7. Hazard Ratios for Study Outcomes by Quartiles of Coefficient of Variation of Body Weight Stratified by Intervention Group in the Look AHEAD Study

	Quartiles of Coefficient of Variation of Body Weight, %				P_{trend}	Per 3%
	< 1.97	1.97-3.15	3.15-4.94	> 4.94		
**Diabetes Support and Education (n = 1775)**						
**All-cause mortality**						
Model 1	Reference	2.18 (1.23-3.84)	1.77 (0.90-3.46)	4.21 (2.27-7.80)	<0.001	1.47 (1.24-1.73)
Model 2	Reference	2.05 (1.16-3.63)	1.81 (0.92-3.55)	4.20 (2.26-7.82)	<0.001	1.50 (1.26-1.78)
Model 3	Reference	2.05 (1.16-3.62)	1.80 (0.92-3.55)	4.12 (2.21-7.68)	<0.001	1.49 (1.25-1.77)
**Cardiovascular death**						
Model 1	Reference	0.72 (0.07-7.94)	4.56 (0.83-25.02)	12.59 (2.51-63.29)	<0.001	1.60 (1.15-2.22)
Model 2	Reference	0.57 (0.05-6.33)	3.58 (0.63-20.38)	11.79 (2.30-60.33)	0.001	1.73 (1.20-2.49)
Model 3	Reference	0.57 (0.05-6.36)	3.83 (0.67-21.73)	15.32 (2.83-82.91)	0.001	1.80 (1.24-2.60)
**Cardiovascular event**						
Model 1	Reference	1.55 (0.98-2.44)	1.17 (0.67-2.06)	1.91 (1.07-3.38)	0.07	1.12 (0.91-1.38)
Model 2	Reference	1.48 (0.93-2.36)	1.21 (0.69-2.14)	2.11 (1.18-3.77)	0.03	1.18 (0.96-1.45)
Model 3	Reference	1.49 (0.94-2.38)	1.22 (0.69-2.16)	2.20 (1.23-3.94)	0.02	1.20 (0.98-1.47)
**Intensive Lifestyle Intervention (n = 1829)**						
**All-cause mortality**						
Model 1	Reference	0.84 (0.37-1.91)	0.75 (0.35-1.58)	0.84 (0.41-1.73)	0.72	1.11 (0.91-1.36)
Model 2	Reference	0.84 (0.36-1.97)	0.76 (0.34-1.68)	1.01 (0.46-2.21)	0.81	1.24 (1.02-1.51)
Model 3	Reference	0.84 (0.36-1.97)	0.76 (0.34-1.68)	1.01 (0.46-2.21)	0.81	1.24 (1.02-1.51)
**Cardiovascular death**						
Model 1	Reference	1.78 (0.18-17.21)	1.86 (0.22-15.67)	2.03 (0.25-16.45)	0.55	1.33 (0.97-1.82)
Model 2	Reference	1.49 (0.15-14.58)	0.94 (0.10-8.89)	1.34 (0.15-12.28)	0.89	1.56 (1.09-2.25)
Model 3	Reference	1.50 (0.15-14.65)	0.92 (0.10-8.64)	1.29 (0.14-11.87)	0.94	1.56 (1.08-2.26)
**Cardiovascular event**						
Model 1	Reference	0.74 (0.39-1.41)	0.68 (0.37-1.23)	0.60 (0.33-1.08)	0.11	0.93 (0.75-1.14)
Model 2	Reference	0.79 (0.39-1.58)	0.78 (0.41-1.50)	0.80 (0.42-1.55)	0.63	1.07 (0.86-1.32)
Model 3	Reference	0.78 (0.39-1.57)	0.78 (0.40-1.49)	0.80 (0.41-1.55)	0.62	1.07 (0.86-1.32)

Data are hazard ratios (95% confidence intervals) unless otherwise indicated.

Model 1 adjusted for age, sex, race/ethnicity.

Model 2 includes variables in model 1 with further adjustment for current smoking, alcohol drinking, use of antihypertensive medications, average systolic blood pressure, average ratio of total to high-density lipoprotein cholesterol, average hemoglobin A_{1C}, estimated glomerular filtration rate, and duration of diabetes.

Model 3 includes model 2 plus further adjustment for average body weight.

AHEAD indicates Action for Health in Diabetes.

© 2022 Kaze AD et al. *JAMA Network Open.*

eTable 8. Hazard Ratios for Study Outcomes by Quartiles of VIM of Body Weight Stratified by Intervention Group in the Look AHEAD Study

	Quartiles of VIM of Body Weight,				P_{trend}	Per 21-unit
	< 13.9	13.9-22.1	22.2-34.5	> 34.5		
**Diabetes Support and Education (n = 1775)**						
**All-cause mortality**						
Model 1	Reference	1.69 (0.96-2.97)	1.85 (0.99-3.43)	3.08 (1.67-5.67)	<0.001	1.45 (1.23-1.70)
Model 2	Reference	1.58 (0.90-2.78)	1.80 (0.97-3.36)	3.12 (1.69-5.75)	<0.001	1.47 (1.24-1.73)
Model 3	Reference	1.58 (0.90-2.78)	1.78 (0.95-3.32)	3.00 (1.61-5.59)	0.001	1.45 (1.23-1.72)
**Cardiovascular death**						
Model 1	Reference	0.72 (0.07-7.98)	5.39 (1.04-27.90)	8.74 (1.68-45.48)	0.002	1.56 (1.14-2.13)
Model 2	Reference	0.56 (0.05-6.19)	4.19 (0.80-22.03)	7.69 (1.46-40.37)	0.003	1.63 (1.16-2.31)
Model 3	Reference	0.54 (0.05-6.08)	4.75 (0.89-25.26)	13.17 (2.22-78.28)	0.002	1.74 (1.22-2.48)
**Cardiovascular event**						
Model 1	Reference	1.74 (1.10-2.74)	1.19 (0.68-2.08)	1.68 (0.93-3.02)	0.15	1.10 (0.90-1.35)
Model 2	Reference	1.59 (1.00-2.53)	1.23 (0.70-2.18)	1.78 (0.99-3.22)	0.09	1.15 (0.94-1.40)
Model 3	Reference	1.61 (1.01-2.56)	1.29 (0.73-2.29)	1.97 (1.08-3.58)	0.05	1.18 (0.97-1.45)
**Intensive Lifestyle Intervention (n = 1829)**						
**All-cause mortality**						
Model 1	Reference	0.97 (0.43-2.24)	0.93 (0.43-2.00)	0.93 (0.43-1.97)	0.81	1.10 (0.89-1.35)
Model 2	Reference	0.99 (0.41-2.37)	0.92 (0.41-2.10)	1.12 (0.50-2.54)	0.73	1.22 (1.00-1.48)
Model 3	Reference	0.99 (0.41-2.37)	0.92 (0.41-2.10)	1.12 (0.50-2.54)	0.73	1.22 (1.00-1.49)
**Cardiovascular death**						
Model 1	Reference	2.30 (0.26-20.74)	2.13 (0.26-17.58)	1.55 (0.18-13.04)	0.95	1.27 (0.91-1.77)
Model 2	Reference	2.25 (0.25-20.56)	0.93 (0.10-8.77)	1.21 (0.13-11.47)	0.71	1.48 (1.02-2.16)
Model 3	Reference	2.27 (0.25-20.79)	0.96 (0.10-9.01)	1.24 (0.13-11.77)	0.73	1.50 (1.02-2.19)
**Cardiovascular event**						
Model 1	Reference	0.81 (0.43-1.52)	0.66 (0.36-1.20)	0.58 (0.32-1.05)	0.06	0.92 (0.75-1.15)
Model 2	Reference	0.89 (0.45-1.74)	0.71 (0.37-1.37)	0.75 (0.39-1.45)	0.33	1.04 (0.84-1.30)
Model 3	Reference	0.89 (0.45-1.74)	0.71 (0.37-1.37)	0.75 (0.39-1.44)	0.33	1.05 (0.84-1.30)

Data are hazard ratios (95% confidence intervals) unless otherwise indicated.

Model 1 adjusted for age, sex, race/ethnicity.

Model 2 includes variables in model 1 with further adjustment for current smoking, alcohol drinking, use of antihypertensive medications, average systolic blood pressure, average ratio of total to high-density lipoprotein cholesterol, average hemoglobin A_{1C}, estimated glomerular filtration rate, and duration of diabetes.

Model 3 includes model 2 plus further adjustment for average body weight.

AHEAD indicates Action for Health in Diabetes; VIM, variability independent of the mean.

© 2022 Kaze AD et al. *JAMA Network Open.*

eTable 9. Hazard Ratios for Study Outcomes by Quartiles of SD of Body Weight Stratified by Intervention Group in the Look AHEAD Study

	Quartiles of SD of Body Weight, kg				P_{trend}	Per 3 kg
	< 1.9	1.9-3.0	3.01-4.7	> 4.7		
**Diabetes Support and Education (n = 1775)**						
**All-cause mortality**						
Model 1	Reference	1.57 (0.86-2.86)	2.50 (1.37-4.57)	3.32 (1.81-6.11)	<0.001	1.42 (1.22-1.65)
Model 2	Reference	1.48 (0.81-2.70)	2.41 (1.32-4.40)	3.25 (1.76-6.00)	<0.001	1.42 (1.22-1.66)
Model 3	Reference	1.48 (0.81-2.70)	2.39 (1.30-4.40)	3.21 (1.70-6.06)	<0.001	1.41 (1.20-1.66)
**Cardiovascular death**						
Model 1	Reference	2.85 (0.26-31.45)	10.40 (1.21-89.42)	14.39 (1.67-124.18)	0.002	1.50 (1.12-2.00)
Model 2	Reference	2.24 (0.20-25.03)	9.18 (1.07-79.07)	10.14 (1.14-90.56)	0.007	1.53 (1.10-2.12)
Model 3	Reference	2.39 (0.21-27.00)	11.93 (1.34-105.76)	18.21 (1.86-178.54)	0.002	1.67 (1.18-2.35)
**Cardiovascular event**						
Model 1	Reference	1.51 (0.96-2.39)	1.05 (0.59-1.85)	1.52 (0.87-2.66)	0.27	1.08 (0.89-1.31)
Model 2	Reference	1.40 (0.88-2.22)	1.09 (0.61-1.92)	1.54 (0.87-2.72)	0.23	1.11 (0.91-1.35)
Model 3	Reference	1.45 (0.91-2.31)	1.21 (0.68-2.17)	1.82 (1.01-3.28)	0.08	1.17 (0.96-1.42)
**Intensive Lifestyle Intervention (n = 1829)**						
**All-cause mortality**						
Model 1	Reference	0.59 (0.25-1.35)	0.94 (0.47-1.90)	0.85 (0.42-1.74)	0.85	1.08 (0.87-1.34)
Model 2	Reference	0.71 (0.29-1.72)	1.09 (0.51-2.34)	1.08 (0.49-2.38)	0.49	1.19 (0.96-1.46)
Model 3	Reference	0.71 (0.29-1.72)	1.10 (0.51-2.36)	1.09 (0.49-2.43)	0.47	1.19 (0.97-1.47)
**Cardiovascular death**						
Model 1	Reference	1.81 (0.19-17.48)	2.29 (0.28-18.77)	2.15 (0.26-17.71)	0.51	1.20 (0.83-1.74)
Model 2	Reference	1.82 (0.18-17.96)	1.37 (0.15-12.18)	1.70 (0.18-16.11)	0.79	1.38 (0.91-2.10)
Model 3	Reference	1.81 (0.18-17.99)	1.45 (0.16-13.06)	1.87 (0.19-18.16)	0.70	1.43 (0.95-2.15)
**Cardiovascular event**						
Model 1	Reference	0.73 (0.39-1.36)	0.65 (0.36-1.16)	0.63 (0.35-1.13)	0.14	0.93 (0.75-1.16)
Model 2	Reference	0.82 (0.42-1.60)	0.73 (0.38-1.38)	0.77 (0.40-1.47)	0.44	1.02 (0.81-1.28)
Model 3	Reference	0.82 (0.42-1.60)	0.73 (0.38-1.38)	0.77 (0.40-1.48)	0.45	1.02 (0.81-1.28)

Data are hazard ratios (95% confidence intervals) unless otherwise indicated.

Model 1 adjusted for age, sex, race/ethnicity.

Model 2 includes variables in model 1 with further adjustment for current smoking, alcohol drinking, use of antihypertensive medications, average systolic blood pressure, average ratio of total to high-density lipoprotein cholesterol, average hemoglobin A_{1C}, estimated glomerular filtration rate, and duration of diabetes.

Model 3 includes model 2 plus further adjustment for average body weight.

AHEAD indicates Action for Health in Diabetes; SD, standard deviation.

© 2022 Kaze AD et al. *JAMA Network Open.*

eTable 10. Hazard Ratios for Study Outcomes by Coefficient of Variation of BMI and Treatment Group After Additional Adjustment for BMI Change

	Quartiles of Coefficient of Variation of BMI, %					Per 3%
	**< 1.97**	**1.97-3.15**	**3.15-4.94**	**> 4.94**	***P* for trend**	
**Diabetes Support and Education (n =1775)**						
**All-cause mortality**	Reference	2.03 (1.15-3.59)	1.77 (0.90-3.49)	3.92 (2.06-7.49)	<0.001	1.56 (1.25-1.94)
**Cardiovascular death**	Reference	0.55 (0.05-6.16)	4.15 (0.71-24.30)	12.34 (2.07-73.40)	0.002	2.25 (1.22-4.15)
**Cardiovascular event**	Reference	1.50 (0.94-2.38)	1.26 (0.72-2.23)	2.33 (1.30-4.17)	0.02	1.33 (1.06-1.68)
**Intensive Lifestyle Intervention (n = 1829)**						
**All-cause mortality**	Reference	0.79 (0.34-1.86)	0.67 (0.30-1.50)	0.70 (0.29-1.69)	0.43	1.16 (0.86-1.56)
**Cardiovascular death**	Reference	1.20 (0.12-11.93)	0.60 (0.06-5.81)	0.39 (0.03-4.79)	0.27	1.24 (0.55-2.79)
**Cardiovascular event**	Reference	0.75 (0.38-1.51)	0.74 (0.38-1.44)	0.75 (0.37-1.53)	0.51	1.07 (0.86-1.33)

Hazard ratios were obtained from Cox proportional hazard models adjusted for age, sex, race/ethnicity, current smoking, alcohol drinking, use of antihypertensive medications, average systolic blood pressure, average ratio of total to high-density lipoprotein cholesterol, average hemoglobin A_{1C}, estimated glomerular filtration rate, and duration of diabetes, average BMI and BMI change between the baseline and fourth visits.

AHEAD indicates Action for Health in Diabetes; BMI, body mass index. * $P<0.05$, † $P<0.01$, ‡ $P<0.001$

© 2022 Kaze AD et al. *JAMA Network Open.*

eTable 11. Hazard Ratios for Study Outcomes by Coefficient of Variation of WC and Treatment Group After Additional Adjustment for WC Change

	Quartiles of Coefficient of Variation of Waist Circumference, %				P_{trend}	Per 2.6%
	< 1.91	1.91-2.94	2.95-4.49	> 4.49		
**Diabetes Support and Education (n = 1775)**						
**All-cause mortality**	Reference	1.00 (0.57-1.76)	1.12 (0.61-2.04)	1.93 (1.05-3.55)	0.07	1.35 (1.05-1.73)
**Cardiovascular death**	Reference	1.99 (0.35-11.13)	1.59 (0.25-10.25)	4.60 (0.67-31.52)	0.19	1.47 (0.61-3.55)
**Cardiovascular event**	Reference	1.12 (0.70-1.78)	0.93 (0.54-1.61)	1.34 (0.75-2.42)	0.55	1.14 (0.89-1.46)
**Intensive Lifestyle Intervention (n = 1829)**						
**All-cause mortality**	Reference	0.97 (0.46-2.03)	0.52 (0.23-1.20)	1.21 (0.58-2.54)	0.76	1.15 (0.89-1.48)
**Cardiovascular death**	Reference	0.45 (0.10-2.12)	0.10 (0.01-0.94)	0.41 (0.09-1.91)	0.22	1.32 (0.76-2.30)
**Cardiovascular event**	Reference	0.42 (0.21- 0.83)	0.76 (0.43-1.34)	0.63 (0.33-1.18)	0.41	1.06 (0.83-1.34)

Hazard ratios were obtained from Cox proportional hazard models adjusted for age, sex, race/ethnicity, current smoking, alcohol drinking, use of antihypertensive medications, average systolic blood pressure, average ratio of total to high-density lipoprotein cholesterol, average hemoglobin A_{1C}, estimated glomerular filtration rate, and duration of diabetes, average WC and WC change between the baseline and fourth visits.

AHEAD indicates Action for Health in Diabetes; WC, waist circumference.

© 2022 Kaze AD et al. *JAMA Network Open.*

eTable 12. Hazard Ratios for Study Outcomes by Coefficient of Variation of BMI and Treatment Group After Restricting Analyses to Participants With Weight Change Less Than 5 kg

	Quartiles of Coefficient of Variation of BMI, %					Per 3%
	< 1.97	1.97-3.15	3.15-4.94	> 4.94	*P* for trend	
**Diabetes Support and Education (n = 1151)**						
**All-cause mortality**	Reference	2.23 (1.17-4.27)	0.80 (0.19-3.48)	9.95 (3.53-28.05)	0.002	2.02 (1.27-3.24)
**Cardiovascular death***	…	…	…	…	…	…
**Cardiovascular event**	Reference	1.75 (1.04-2.97)	0.42 (0.10-1.75)	4.92 (1.72-14.13)	0.12	1.52 (0.90-2.56)
**Intensive Lifestyle Intervention (n = 864)**						
**All-cause mortality**	Reference	0.86 (0.30-2.49)	0.68 (0.21-2.24)	1.40 (0.33-5.89)	0.97	0.94 (0.40-2.23)
**Cardiovascular death***	…	…	…	…	…	…
**Cardiovascular event**	Reference	0.61 (0.26-1.42)	0.49 (0.18-1.29)	1.01 (0.34-3.04)	0.51	0.80 (0.39-1.62)

Hazard ratios were obtained from Cox proportional hazard models adjusted for age, sex, race/ethnicity, current smoking, alcohol drinking, use of antihypertensive medications, average systolic blood pressure, average ratio of total to high-density lipoprotein cholesterol, average hemoglobin A_{1C}, estimated glomerular filtration rate, and duration of diabetes, average BMI and BMI change between the baseline and fourth visits. * Only 4 cardiovascular deaths were observed in this group, hence hazard ratios for cardiovascular deaths could not be computed. AHEAD indicates Action for Health in Diabetes; BMI, body mass index.

© 2022 Kaze AD et al. *JAMA Network Open.*

eTable 13. Hazard Ratios for Study Outcomes by Coefficient of Variation of Waist Circumference and Treatment Group After Restricting Analyses to Participants With Weight Change Less Than 5 kg

	**Quartiles of Coefficient of Variation of Waist Circumference, %**				**P_{trend}**	**Per 2.6%**
	**< 1.91**	**1.91-2.94**	**2.95-4.49**	**> 4.49**		
**Diabetes Support and Education (n = 1151)**						
**All-cause mortality**	Reference	0.94 (0.45-1.99)	1.13 (0.49-2.62)	1.95 (0.75-5.13)	0.28	1.38 (0.85-2.22)
**Cardiovascular death***	…	…	…	…	…	…
**Cardiovascular event**	Reference	1.09 (0.62-1.91)	0.81 (0.39-1.68)	0.52 (0.15-1.76)	0.32	0.79 (0.49-1.28)
**Intensive Lifestyle Intervention (n = 864)**						
**All-cause mortality**	Reference	1.55 (0.50-4.78)	0.98 (0.25-3.81)	2.03 (0.48-8.57)	0.53	1.27 (0.81-1.99)
**Cardiovascular death***	…	…	…	…	…	…
**Cardiovascular event**	Reference	0.25 (0.08-0.80)	1.23 (0.57-2.66)	0.42 (0.10-1.70)	0.73	0.73 (0.41-1.29)

Hazard ratios were obtained from Cox proportional hazard models adjusted for age, sex, race/ethnicity, current smoking, alcohol drinking, use of antihypertensive medications, average systolic blood pressure, average ratio of total to high-density lipoprotein cholesterol, average hemoglobin A_{1C}, estimated glomerular filtration rate, and duration of diabetes, average WC and WC change between the baseline and fourth visits. * Only 4 cardiovascular deaths were observed in this group, hence hazard ratios for cardiovascular deaths could not be computed.

AHEAD indicates Action for Health in Diabetes; WC, waist circumference.

© 2022 Kaze AD et al. *JAMA Network Open.*
